# Effects of the Pattern of Light and a Dark Recovery Period on Photosynthesis and Growth in *Synechocystis* sp. PCC 6803

**DOI:** 10.3390/microorganisms14092044

**Published:** 2026-09-14

**Authors:** Hyun-Jin Lim, Yeong-Jun Lee, Min-Sung Kim, Matthias Rögner, Jong-Hee Kwon

**Affiliations:** 1 Division of Applied Life Sciences (BK21), Gyeongsang National University, Jinju 52828, Republic of Korea; nirvana970311@gmail.com (H.-J.L.); log1124k@naver.com (Y.-J.L.); w1star@daum.net (M.-S.K.); 2 Department of Food Science & Technology and Institute of Agriculture & Life Science, Gyeongsang National University, Jinju 52828, Republic of Korea; 3 Plant Biochemistry, Faculty of Biology & Biotechnology, Ruhr University Bochum, D-44780 Bochum, Germany

**Keywords:** cyanobacteria, turbidostat cultivation, light/dark cycle, oxygen evolution, photoinhibition

## Abstract

Optimizing the application of light is a central challenge in the cultivation of cyanobacteria because light is needed for growth, but excessive light can cause cellular damage. Here, we demonstrate that the temporal pattern of the light intensity, rather than the time-integrated daily total, determines photosynthetic stability and growth in *Synechocystis* sp. PCC 6803. Cells were cultivated in a turbidostatic flat-panel photobioreactor using three different light patterns, however, with identical time-integrated daily total (3.19 × 10^7^ µmol photons m^−2^ day^−1^): a simulated solar light/dark (L/D) pattern, with increasing and then decreasing light intensity during the light period; a ‘rectangular’ L/D pattern, with a constant light intensity during the light period; and a constant light pattern, with no dark period. The rectangular L/D pattern led to the most stable and productive photosynthesis: 7161.3 ± 321.2 µmol O_2_ L^−1^ day^−1^ and the volumetric biomass productivity (0.072 ± 0.002 g DCW L^−1^ day^−1^); these values were approximately 1.2-fold higher than those under the simulated solar L/D pattern. In contrast, constant light intensity at 370 µmol photons m^−2^ s^−1^ initially increased O_2_ evolution, but photosynthetic activity decreased over time. A constant light intensity of 150 µmol photons m^−2^ s^−1^ prevented this collapse but also decreased O_2_ evolution. During the light period, Western blotting of the D1 protein of PSII indicated damage to this protein and measurements of chlorophyll content indicated pigment-related stress. Cells recovered from this damage during the dark period. These findings show that recovery during a dark period was required for the sustained photosynthesis of *Synechocystis* sp. PCC 6803 under conditions of continuous cultivation and that the rectangular L/D pattern was the most effective of the three temporal patterns.

## 1. Introduction

Photosynthetic microorganisms, especially microalgae and cyanobacteria, have the potential for a sustainable production of biomass, biofuels, and value-added compounds [1]. Cyanobacteria are photoautotrophic microorganisms that can produce renewable energy carriers, such as biohydrogen and biodiesel, which are potential alternatives for future energy systems [2,3]. As photosynthesis drives the growth of microalgae and cyanobacteria, the optimization of light conversion into cellular biomass can improve the efficiency and increase the productivity of phototrophic cultivation systems [4].

However, maximizing the productivity of photosynthetic microorganisms is not as simple as just increasing the light intensity, because these organisms can adapt to changes in the wavelength and intensity of light [5]. Many studies have attempted to determine the optimal level of light to improve overall photosynthetic performance. For example, photosynthetic productivity can be enhanced by optimizing light conditions, including changes in light wavelength, such as the use of red light [5,6,7], as well as adjustments in light pattern [8,9]. However, when the level of light exceeds the photosynthetic capacity of cells, it can decrease productivity and even cause irreversible cellular damage [10,11,12]. Therefore, an efficient strategy for increasing photosynthesis and decreasing cell damage is required.

The light experienced by cells in dense algal or cyanobacterial cultures depends on the incident light, cell density, culture depth, and mixing conditions [13]. While a low light level results in decreased photosynthesis and cell growth, even a moderate light level can induce photodamage, especially of photosystem II (PSII), with the damage increasing with increasing light levels [12,14]. Within PSII, the D1 protein (also referred to as PsbA) is highly sensitive to light-induced oxidative degradation, and damaged D1 must be repaired or replaced to maintain photosynthetic electron transport [15]. Although photosynthetic organisms have evolved various photoprotective mechanisms, photodamage can exceed their capacity under very high intensities [16,17,18].

Outdoor-grown photosynthetic organisms are exposed to large daily changes in light level, with transient periods of excessive light during daytime [19] decreasing photosynthetic efficiency due to photoinhibition. Cyanobacteria also exhibit circadian rhythms [20,21], and their metabolism in natural ecosystems is therefore very different during the day and night [22,23]. In particular, the dark period may enable the cells to repair photodamaged photosynthetic components, including the D1 protein [24,25].

Regarding light-use efficiency, it is important to distinguish between time-integrated light and the temporal pattern by which the light is delivered: For a single time-integrated daily level of light, photosynthetic productivity and cell growth can depend on whether the light is applied as a slowly increasing and then decreasing level during the ‘day’, followed by a period of darkness. Alternatively, a period of constant light during the ‘day’ could be followed by a period of darkness, or there could be a constant low level of light without darkness [26]. A light/dark (L/D) regimen with a constant daytime light level may result in decreased photodamage and efficient recovery of damage during the dark period [27,28]. In contrast, even low-level constant light may limit damage recovery and decrease long-term photosynthetic stability [26,29].

*Synechocystis* sp. PCC 6803 is a well-characterized model cyanobacterium for the study of photosynthesis, photophysiology, and bioprocess optimization [30,31,32]. Its genetic tractability and metabolic versatility have also made it an important chassis for the development of light-driven cyanobacterial cell factories [30,33]. Recent studies have demonstrated that controlling light availability in *Synechocystis* cultures can substantially affect photosynthetic performance, cell growth, and biomass productivity [34,35]. Moreover, its photosynthetic responses can be quantitatively evaluated through oxygen evolution and changes in the D1 protein, providing information on photosystem II activity, photodamage, and recovery [36,37]. Therefore, *Synechocystis* sp. PCC 6803 provides a suitable model for investigating the effects of temporal light patterns, and such knowledge may contribute to optimizing illumination strategies and improving biomass productivity in cyanobacterial cultivation systems [33,38].

In the present study, we cultivated *Synechocystis* sp. PCC 6803 under three different temporal light patterns with identical time-integrated daily light intensity: (i) a simulated solar L/D pattern, with a slow increase and later decrease in light intensity during daytime, followed by a period of darkness; (ii) a ‘rectangular’ L/D pattern, in which there was a period of constant light intensity followed by a period of darkness; and (iii) a constant light intensity pattern, with no dark period. Maintaining the same time-integrated daily light intensity enabled the evaluation of the effects of these different temporal patterns on photosynthesis over time, including total daily photosynthesis, effective growth rate, and abundance of the D1 protein.

## 2. Materials and Methods

### 2.1. Photobioreactor

A flat-panel photobioreactor with a working volume of 5 L was used for continuous cultivation. The reactor was a flat, rectangular container with front and back windows and had a light-path of 40 mm, as shown in Supplementary Figure S1. The light source consisted of two LED panels that produced white light–one on the front and one on the back. The same LED light source was used for all illumination experiments. The light intensity was determined by a Li-Cor LI-189 2 π PAR quantum sensor.

Cell density was controlled by a turbidostatic procedure using a real-time turbidity sensor (Trb 8300, Mettler-Toledo GmbH, Urdorf, Switzerland). The turbidity set point was fixed at 28%, corresponding to an OD_750_ of 2.0. Upon exceeding this set point, fresh medium was supplied by a peristaltic pump while an equal volume of culture was simultaneously removed. Dilution was stopped once the turbidity decreased below 28%. The culture was continuously aerated at a gas flow rate of 50 L min^−1^ with air containing 3% (*v*/*v*) CO_2_, and the culture pH was maintained at 7.0. All experimental parameters (cell turbidity, pH, light intensity, and medium flow rate) were controlled and monitored by process automation programming using LabVIEW software. Prior to cultivation, the reactor system was chemically sterilized by incubation with a 5 mM peroxyacetic acid solution for 2 h, followed by washing twice with sterile water.

### 2.2. Strain and Cultivation Conditions

**Figure 1:**
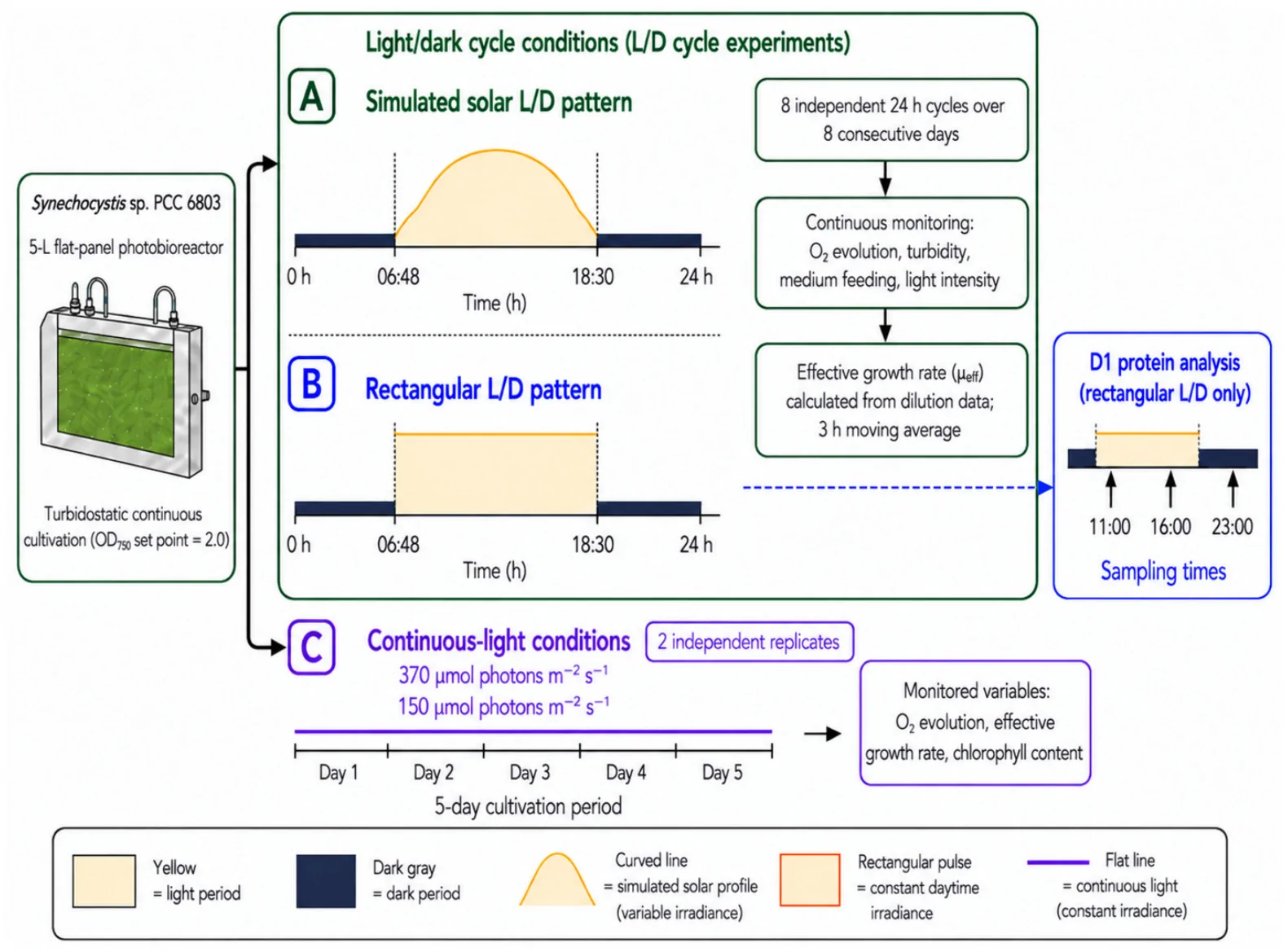
Schematic overview of the experimental design.

*Synechocystis* sp. PCC 6803 wild type from the Pasteur Culture Collection, hereinafter referred to as *Synechocystis*, was cultivated in YBG11 medium (1.76·10^−2^ M NaNO_3_; 1·10^−2^ M HEPES; 1.75·10^−3^ M K_2_HPO_4_; 3·10^−4^ M MgSO_4_; 1.8·10^−4^ M Na_2_CO_3_; 1.6·10^−5^ M Na_2_-EDTA; 4.5·10^−5^ M Boric acid; 3·10^−5^ M Citric acid; 9·10^−6^ M MnCl_2_; 6·10^−6^ M FeCl_3_; 1.6·10^−6^ M Na_2_MoO_4_; 7·10^−7^ M ZnSO_4_; 3·10^−7^ M CuSO_4_; 1.6·10^−7^ M Co(NO_3_)_2_; 2.5·10^−4^ M CaCl_2_) [39]. Cultivation was performed using a turbidostat with a standard set point (OD_750_ = 2.0). To evaluate the effect of the temporal pattern of light intensity on photosynthesis and growth, three different patterns were applied (Figure 1): (i) a simulated solar L/D pattern, with the light intensity changing during the ‘day’, followed by a dark period; (ii) a rectangular L/D pattern, with a period of constant light intensity followed by a period of darkness; and (iii) a constant light intensity pattern, with no dark period. The simulated solar L/D pattern mimicked daily data of light intensity of sunlight from the Institute of Energy Systems and Energy Economics, Ruhr University, Germany. Due to the limited capacity of the used LED panels, the solar profile was scaled by a factor of 0.32, resulting in a maximum light intensity of 1400 µmol photons m^−2^ s^−1^. For the simulated solar L/D pattern, the light intensity was adjusted at 0.1 h intervals (based on data from the Institute of Energy Systems and Energy Economics) and was administered from 06:48 to 18:30, corresponding to a ‘daytime’ duration of 11.7 h.

The rectangular L/D pattern had the same ‘daytime’ duration (11.7 h) but with a constant light intensity of 758 µmol photons m^−2^ s^−1^ during the light period. In contrast, cells with a constant light intensity pattern were exposed to a light intensity of 370 µmol photons m^−2^ s^−1^ in one group. Notably, all three patterns provided the identical time-integrated daily total of 3.19 × 10^7^ µmol photons m^−2^ day^−1^. An additional continuous-light condition at 150 µmol photons m^−2^ s^−1^ provided a lower daily photon dose of 1.30 × 10^7^ µmol photons m^−2^ day^−1^.

### 2.3. Determination of Oxygen Evolution and Effective Growth Rate

Oxygen evolution and effective growth rate were monitored during turbidostatic continuous cultivation acc. to Kwon et al. [40], with minor modifications. For the illumination conditions involving light/dark (L/D) cycles, eight independent 24 h experimental cycles were conducted over eight consecutive days. In contrast, the continuous-light experiments at 370 or 150 µmol photons m^−2^ s^−1^ were conducted for 5 days, during which temporal changes in oxygen evolution, effective growth rate, and chlorophyll content were monitored.

The rate of oxygen evolution during cultivation was measured continuously using a BCP-O_2_ sensor (BlueSens GmbH, Regensburg, Germany), which was directly connected to the exhaust-gas outlet line of the photobioreactor. The determined O_2_-evolution was used as an indicator of photosynthetic productivity, because it reflects the balance between the photosynthetic evolution of O_2_ by water splitting in PSII and the cellular O_2_-consumption by respiration and other reactions.

The effective growth rate (µ_eff_) was determined from the dilution data recorded during turbidostatic cultivation, as described by Kwon et al. [40]. Briefly, µ_eff_ was calculated by multiplying the fixed dilution rate by the dilution frequency recorded over a defined period, thereby allowing quantitative estimation of the growth of *Synechocystis* while maintaining a constant cell density. All process variables, including turbidity, medium feeding, light intensity, and oxygen evolution, were continuously recorded using an online control system operated with LabVIEW software. Additionally, for the analysis of short-term growth response, a 3 h moving average was applied to µ_eff_ to visualize temporal changes.

### 2.4. Pigment Analysis

Chlorophyll *a* content was determined spectrophotometrically after extraction with methanol. An aliquot of the *Synechocystis* culture equivalent to 1 mL at an OD_750_ of 2.0 was collected by centrifugation at 16,000× *g* for 10 min. After removal of the supernatant, the cell pellet was resuspended in 1.0 mL of 100% methanol and thoroughly vortexed. The suspension was incubated for 15 min at room temperature to extract the photosynthetic pigments and subsequently centrifuged at 16,000× *g* for 10 min. The absorbance of the resulting supernatant was measured at 665 and 652 nm using a UV/Vis spectrophotometer DU 7400 (Shimadzu, Kyoto, Japan). Chlorophyll *a* concentration was calculated according to the equation of Pohland et al. [41].

### 2.5. Protein Extraction and Western Blot Analysis

Light-induced changes in the D1 protein during the rectangular L/D pattern were examined by collecting cells at three different times (11:00, 16:00, and 23:00). The harvested cells were washed twice with 20 mM HEPES buffer (pH 8.0) to remove residual medium and then resuspended in 20 mL of the same buffer. Cells were disrupted by passing through a French press (1200 psi) three times. Proteins were separated by sodium dodecyl sulfate–polyacrylamide gel electrophoresis (SDS-PAGE) using a discontinuous gel system with a 14% resolving gel. Equal amounts of protein (4 μg per lane) were loaded and electrophoresed at 130 V. The separated proteins were transferred onto a polyvinylidene fluoride (PVDF) membrane by semi-dry blotting at 20 V and 54 mA for 45 min. After overnight blocking, the membrane was incubated with an anti-D1 primary antibody (Agrisera, AS10 704; 1:2500) for 1 h, washed three times with Tris-buffered saline containing Tween 20 (TBST), and then incubated with an alkaline phosphatase-conjugated secondary antibody (Sigma-Aldrich, A3687, St. Louis, MO, USA) for 1 h. After three additional washes with TBST, D1 protein bands were detected using nitro blue tetrazolium (NBT) and 5-bromo-4-chloro-3-indolyl phosphate (BCIP) as substrates for alkaline phosphatase [42].

### 2.6. Statistical Analysis

Data were analyzed using Statistical Analysis System software SAS version 9.4 for Windows (SAS Institute, Inc., Cary, NC, USA). Group differences were determined by a one-way ANOVA followed by Duncan’s test. A *p*-value below 0.05 was considered significant.

## 3. Results

### 3.1. Effects of the Temporal Pattern of Light Intensity on Photosynthetic Activity

*Synechocystis* cells were cultivated under turbidostatic conditions at a set point corresponding to an OD_750_ of 2.0 to compare the effects of different temporal patterns of light intensity on photosynthetic performance and cell growth (Figure 1). The simulated solar L/D pattern, rectangular L/D pattern, and constant light pattern at 370 µmol photons m^−2^ s^−1^ provided the same time-integrated daily light input of 3.19 × 10^7^ µmol photons m^−2^ day^−1^.

**Figure 2:**
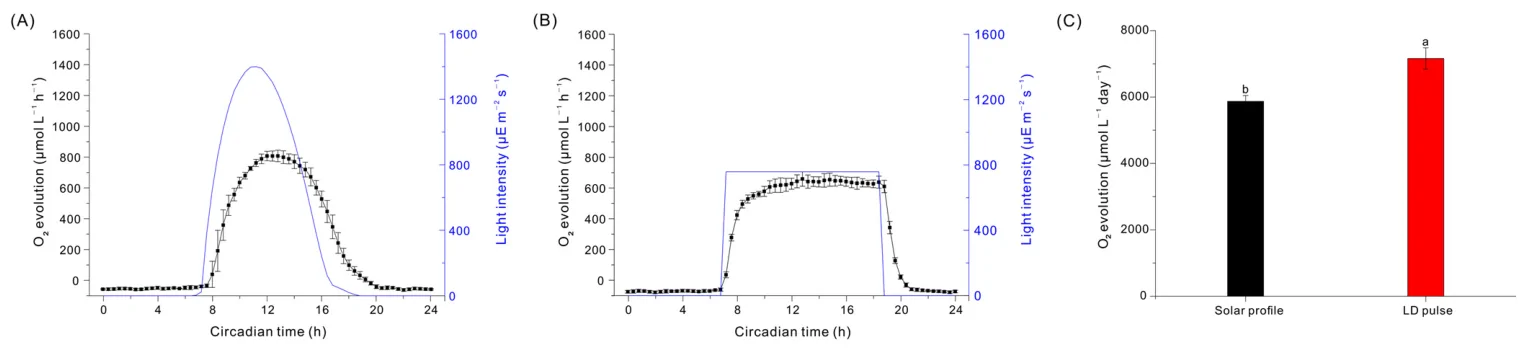
O_2_ evolution of *Synechocystis* under (**A**) the simulated solar L/D pattern and (**B**) the rectangular L/D pattern during turbidostatic cultivation. (**C**) Daily O_2_ evolution under the simulated solar L/D and rectangular L/D patterns. Blue lines indicate the applied light intensity. The data were analyzed using one-way ANOVA, followed by Duncan’s test (*p* < 0.05). Lowercase letters indicate statistically significant differences among groups (*n* = 8).

Under the simulated solar L/D pattern, O_2_ evolution generally followed the daily changes in light intensity with a slight temporal delay (Figure 2A). O_2_ evolution increased after the onset of illumination and reached a maximum of 806.9 ± 30.7 µmol L^−1^ h^−1^ shortly after the light intensity reached its maximum of 1400 µmol photons m^−2^ s^−1^. Thereafter, O_2_ evolution gradually decreased as the light intensity declined. During the dark period, oxygen evolution was close to zero and occasionally negative due to respiration.

Under the rectangular L/D pattern, O_2_ evolution increased rapidly after the onset of illumination and gradually reached a relatively stable level of approximately 600–650 µmol L^−1^ h^−1^, which was maintained throughout most of the light period (Figure 2B). Following the transition to darkness, O_2_ evolution rapidly decreased to near zero and subsequently became slightly negative. Although the maximum instantaneous O_2_ evolution was lower under the rectangular L/D pattern than under the simulated solar L/D pattern, the daily O_2_ evolution was significantly higher under the rectangular L/D pattern, reaching 7161.3 ± 321.2 µmol L^−1^ day^−1^ compared with 5871.1 ± 175.6 µmol L^−1^ day^−1^ under the simulated solar L/D pattern (Figure 2C).

**Figure 3:**
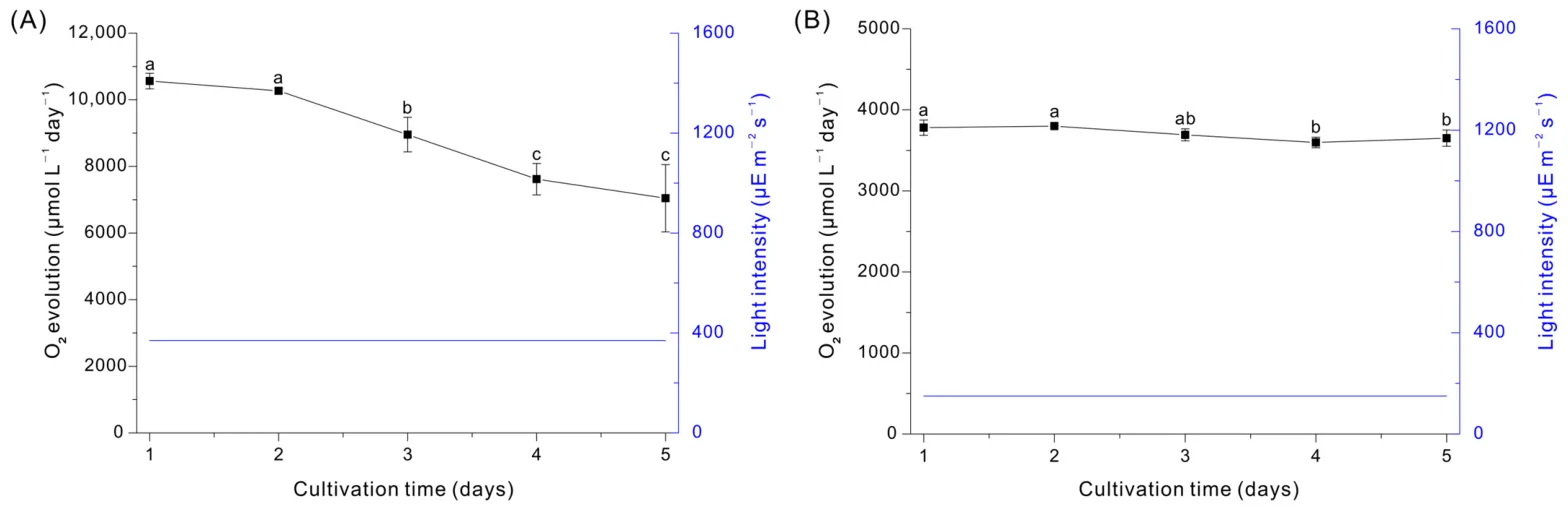
Changes in daily O_2_ evolution of *Synechocystis* during continuous cultivation under constant light conditions. Cells were continuously illuminated at (**A**) 370 µmol photons m^−2^ s^−1^ or (**B**) 150 µmol photons m^−2^ s^−1^. Blue lines indicate the applied light intensity. The data were analyzed using one-way ANOVA, followed by Duncan’s test (*p* < 0.05). Lowercase letters indicate statistically significant differences among groups (*n* = 2).

Continuous illumination at 370 µmol photons m^−2^ s^−1^ initially resulted in high daily O_2_ evolution of 10,561.2 ± 233.3 µmol L^−1^ day^−1^; however, O_2_ evolution progressively decreased during cultivation, reaching 7440.5 ± 1010.1 µmol L^−1^ day^−1^ by day 5 (Figure 3A). In contrast, continuous illumination at 150 µmol photons m^−2^ s^−1^ maintained relatively stable daily O_2_ evolution, ranging from approximately 3600 to 3800 µmol L^−1^ day^−1^ throughout the cultivation period (Figure 3B).

**Figure 4:**
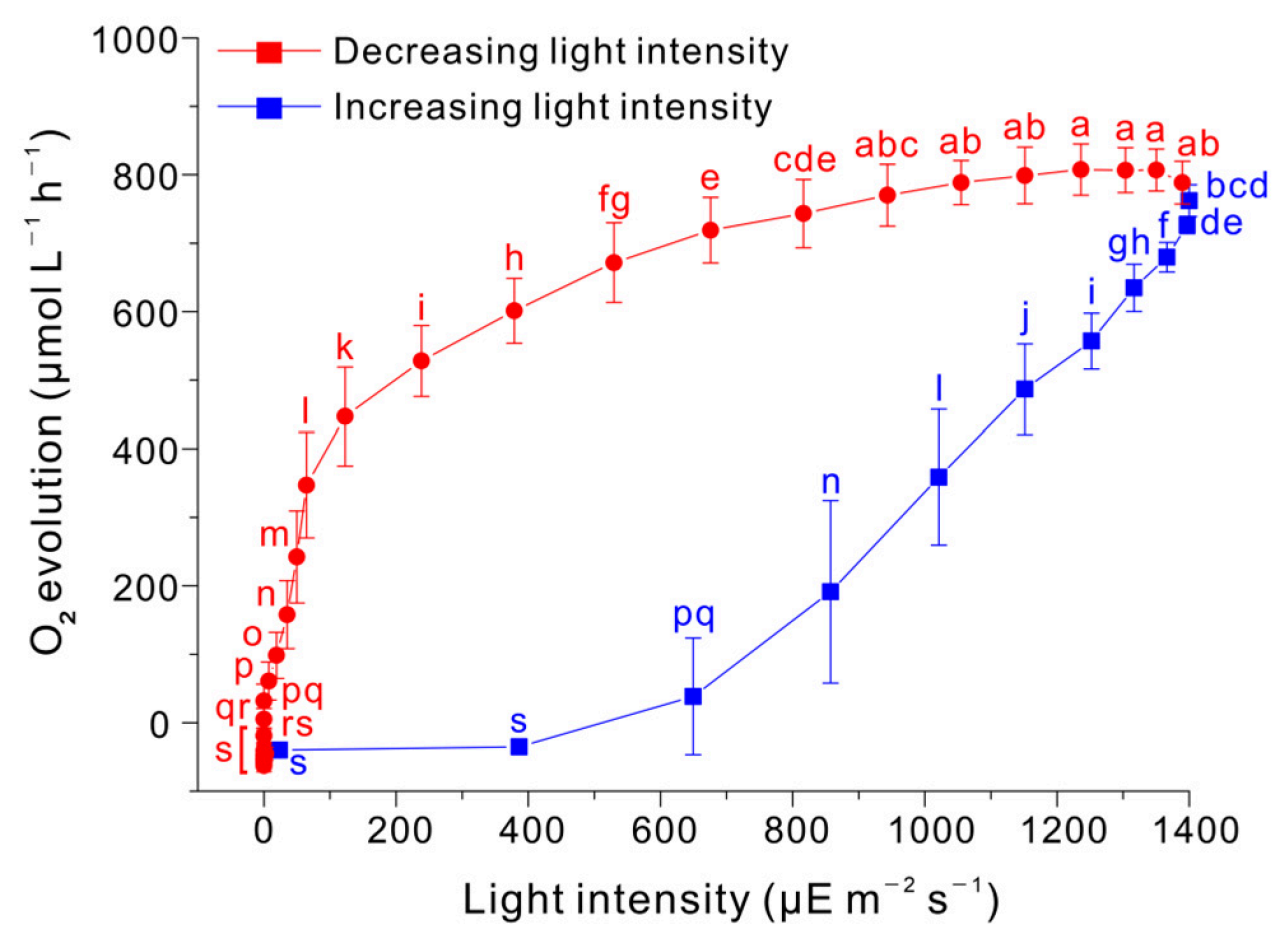
Relationship between light intensity and O_2_ evolution in *Synechocystis* under the simulated solar light/dark (L/D) pattern. Red circles represent the decreasing-light phase, and blue squares represent the increasing-light phase. The data were analyzed using one-way ANOVA, followed by Duncan’s test (*p* < 0.05). Lowercase letters indicate statistically significant differences among groups (*n* = 8).

Plotting O_2_ evolution against light intensity revealed a pronounced hysteresis-like relationship between the increasing- and decreasing-light phases of the simulated solar L/D pattern. At comparable light intensities, O_2_ evolution was consistently higher during the decreasing-light phase, particularly at low to moderate irradiance (Figure 4).

### 3.2. Effects of the Temporal Pattern of Light Intensity on Growth

**Figure 5:**
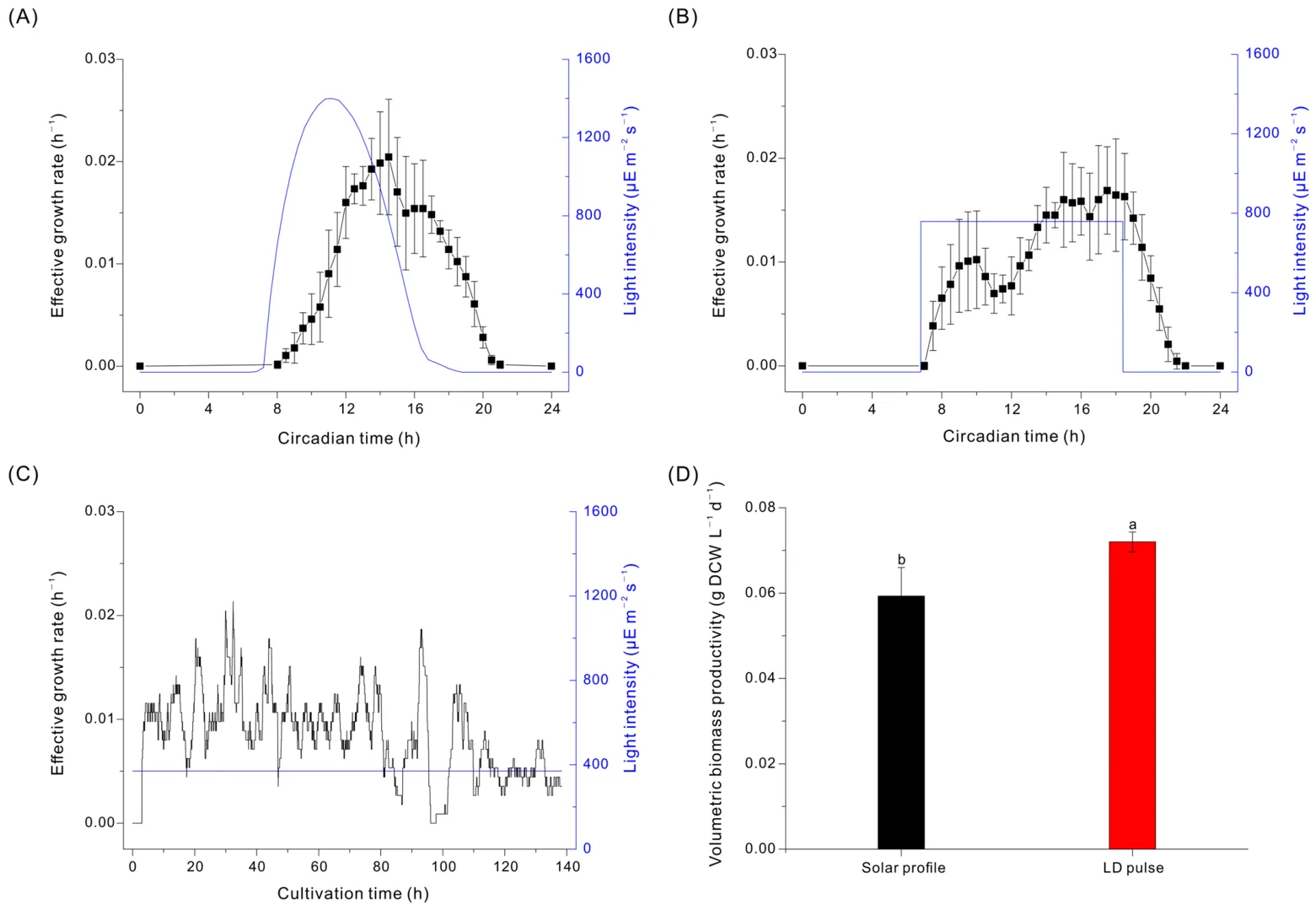
Effective growth rate and volumetric biomass productivity of *Synechocystis* under different light conditions during turbidostatic cultivation. The µ_eff_ was measured under (**A**) the simulated solar light/dark (L/D) pattern, (**B**) the rectangular L/D pattern, and (**C**) continuous illumination at 370 µmol photons m^−2^ s^−1^. Blue lines indicate the applied light intensity. (**D**) Volumetric biomass productivity under the simulated solar L/D and rectangular L/D patterns. The data were analyzed using one-way ANOVA, followed by Duncan’s test (*p* < 0.05). Lowercase letters indicate statistically significant differences among groups (*n* = 6).

The effective growth rate (µ_eff_) under the simulated solar L/D pattern increased after the onset of illumination and generally followed the daily light profile with a temporal delay. The µ_eff_ reached a maximum of 0.0204 ± 0.0057 h^−1^ during the light period and subsequently decreased as the light intensity declined, approaching zero during the dark period (Figure 5A). In the rectangular L/D pattern, µ_eff_ increased rapidly after illumination and remained relatively high during the latter part of the light period, reaching 0.0169 ± 0.0042 h^−1^ before decreasing rapidly after the transition to the dark period (Figure 5B).

Continuous illumination at 370 µmol photons m^−2^ s^−1^ caused substantial fluctuations in µ_eff_ during cultivation, with an overall decline during the later cultivation period (Figure 5C). Despite the lower maximum instantaneous µ_eff_, the rectangular L/D pattern resulted in significantly greater volumetric biomass productivity than the simulated solar L/D pattern, with 0.072 ± 0.002 and 0.059 ± 0.007 g DCW L-1 day^−1^, respectively (Figure 5D).

### 3.3. Changes in D1 Protein and Chlorophyll Content

**Figure 6:**
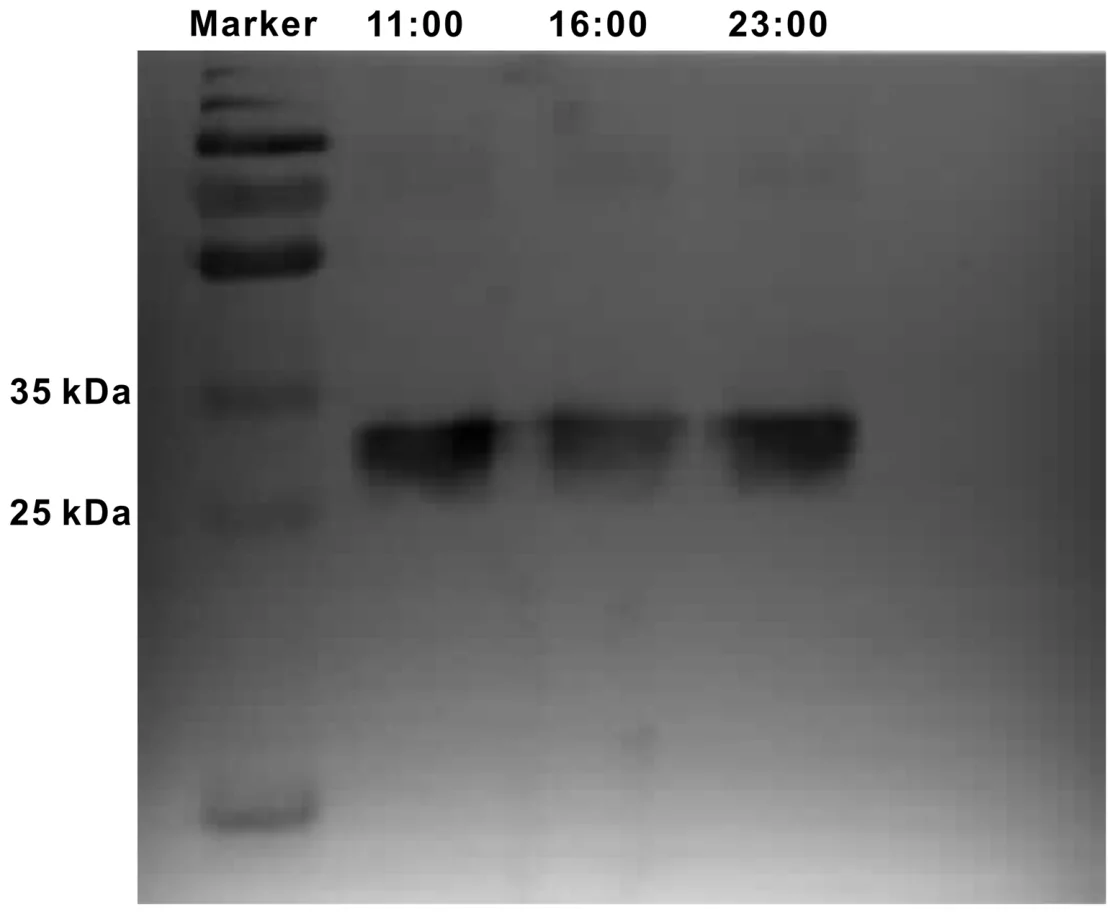
Western blotting of D1 during the light period (11:00 and 16:00) and during the dark period (23:00) of cells grown under the rectangular L/D pattern.

Changes in the D1 protein were examined during cultivation under the rectangular light/dark (L/D) pattern (Figure 6). A clear D1 protein band was detected at 11:00, 4 h after the onset of illumination. The D1 band intensity decreased markedly at 16:00, 2.5 h before the end of the light period. Following the transition to darkness, the D1 band intensity increased again at 23:00, indicating recovery of D1 during the dark period.

**Figure 7:**
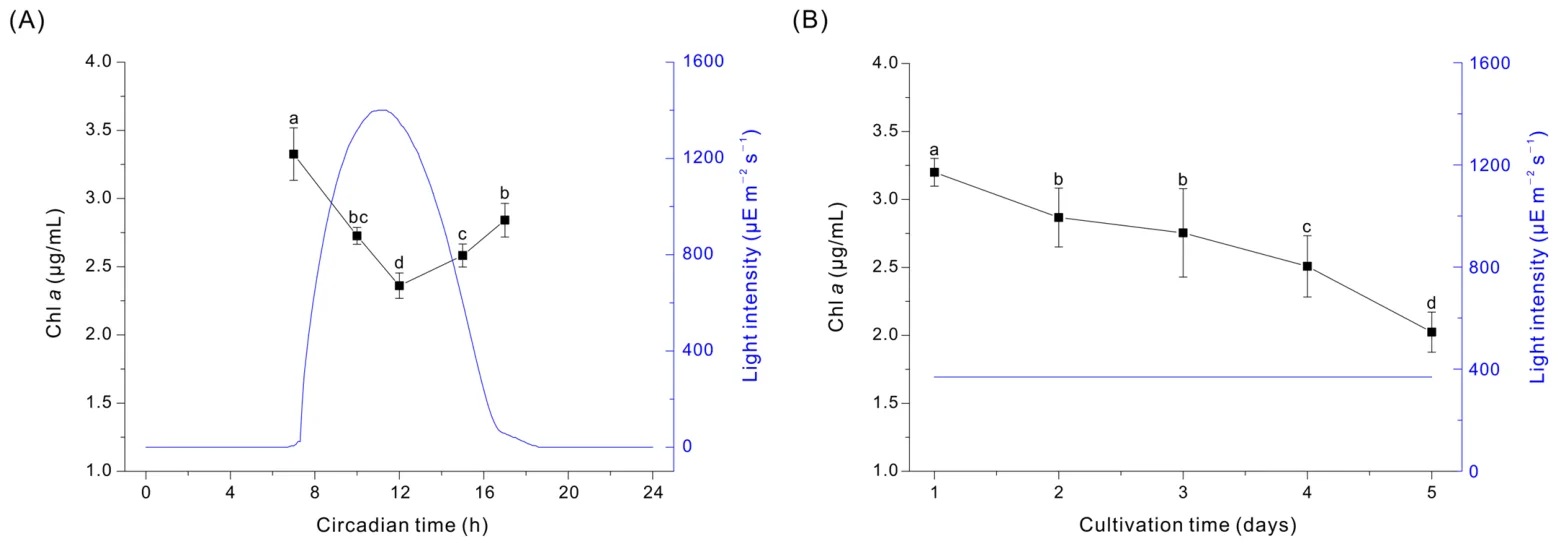
Changes in chlorophyll *a* content of *Synechocystis* under (**A**) the simulated solar light/dark (L/D) pattern and (**B**) continuous illumination at 370 µmol photons m^−2^ s^−1^. The data were analyzed using one-way ANOVA, followed by Duncan’s test (*p* < 0.05). Lowercase letters indicate statistically significant differences among groups (*n* = 3).

Chlorophyll *a* content showed distinct changes in response to the light conditions (Figure 7). During the simulated solar L/D pattern, chlorophyll *a* decreased from 3.33 ± 0.19 µg mL^−1^ in the early light period to a minimum of 2.36 ± 0.09 µg mL^−1^ near the period of maximum light intensity. As the light intensity subsequently decreased, chlorophyll *a* increased again, reaching 2.84 ± 0.12 µg mL^−1^ toward the end of the light period (Figure 7A). In contrast, continuous illumination at 370 µmol photons m^−2^ s^−1^ resulted in a progressive decline in chlorophyll *a* over the cultivation period, decreasing from 3.20 ± 0.10 µg mL^−1^ on day 1 to 2.02 ± 0.14 µg mL^−1^ on day 5 (Figure 7B).

## 4. Discussion

Our results showed that the temporal pattern of the light intensity affects photosynthetic performance in *Synechocystis* when cells are grown under turbidostatic conditions with continuous cultivation. Although cells grown under the simulated solar L/D pattern, the rectangular L/D pattern, and the constant light pattern received the same time-integrated light intensity, these patterns produced distinct physiological responses. The rectangular L/D pattern led to the most stable photosynthesis and the highest volumetric biomass productivity, indicating that a period of constant light intensity followed by a dark period enabled more efficient photosynthesis and accumulation of biomass than the simulated solar L/D pattern.

The present results demonstrate that photosynthetic performance in *Synechocystis* is determined not only by the time-integrated amount of light supplied but also by its temporal distribution. Although the simulated solar L/D pattern, rectangular L/D pattern, and continuous-light condition at 370 µmol photons m^−2^ s^−1^ received the same daily photon input, they produced markedly different photosynthetic and growth responses. In particular, the rectangular L/D pattern provided more stable O_2_ evolution and a higher effective growth rate than the simulated solar L/D pattern, whereas continuous illumination resulted in a progressive loss of photosynthetic performance.

The superior performance of the rectangular L/D pattern may be related to avoidance of the transient high irradiance experienced under the simulated solar profile. In particular, Cordara et al. reported an increase in growth and photosynthetic activity of *Synechocystis* with increasing light up to an optimum, and photoinhibition under excessive light [43]. In the present study, the simulated solar profile reached 1400 µmol photons m^−2^ s^−1^, whereas the rectangular L/D pattern supplied a constant irradiance of 758 µmol photons m^−2^ s^−1^ during the light period. Thus, distributing the same daily photon input at a moderate and constant irradiance may reduce exposure to transient excessive light while maintaining a sufficient photon supply for photosynthesis.

The hysteresis-like relationship between irradiance and O_2_ evolution under the simulated solar profile further suggests that photosynthetic activity was not determined solely by instantaneous light intensity. O_2_ evolution at a given irradiance was greater during decreasing light than during increasing light, particularly at low to moderate irradiances. This difference is consistent with the possibility that the photosynthetic response is influenced by the recent history of light exposure and could be associated with changes in excitation pressure, photosynthetic acclimation, the redox state of the electron transport chain, and photodamage–repair processes. Previous photobioreactor studies have similarly demonstrated that photosynthetic performance under dynamically changing irradiance depends on both the current and preceding light conditions [44]. The rapid establishment of relatively stable O_2_ evolution under the rectangular L/D pattern therefore indicates that constant daytime irradiance provides a more uniform physiological environment than a continuously changing solar-like irradiance.

The importance of the dark period was particularly evident from the continuous-light experiments. Despite receiving the same daily photon input as the two principal L/D patterns, cells exposed continuously to 370 µmol photons m^−2^ s^−1^ exhibited a progressive decline in O_2_ evolution and effective growth rate. Singh et al. similarly reported that a 16 h L/8 h D regimen enhanced growth, photosynthetic pigment accumulation, protein content, and photosynthetic efficiency in *Synechocystis* compared with constant illumination, whereas constant light increased oxidative stress [45]. These findings support the interpretation that the absence of darkness allows light-induced physiological stress to accumulate over successive cultivation periods.

Dark intervals may contribute to photosynthetic stability through several mechanisms. Sforza et al. demonstrated that appropriately adjusted L/D cycles improved photosynthetic efficiency in photobioreactors by allowing re-oxidation of components of the photosynthetic electron transport chain and reducing damage associated with excessive excitation [46]. In addition, diurnal metabolic modeling of *Synechocystis* has indicated that cellular metabolism is temporally partitioned between light-dependent anabolic processes and dark-dependent catabolic processes, during which previously accumulated carbon reserves support cellular metabolism [44]. Darkness should therefore not be regarded simply as a period during which growth stops; rather, it can contribute to metabolic rebalancing and preparation for the subsequent light period.

The temporal changes in D1 abundance may be consistent with this light-induced damage and recovery process. D1 is a core PSII protein that is particularly susceptible to photo-oxidative damage and undergoes continuous degradation and replacement during the PSII repair cycle. Allakhverdiev and Murata reported that the rate of PSII photodamage in *Synechocystis* increases with irradiance, whereas repair requires de novo protein synthesis, including synthesis of D1 [47]. FtsH proteases also have an important role in degradation and replacement of damaged D1 during PSII repair [48]. In the present study, the decreased D1 abundance after prolonged illumination and its subsequent increase during darkness are consistent with dynamic turnover of the D1 protein during recovery of PSII.

The decline in D1 abundance was accompanied by a decrease in the chl *a* content during prolonged illumination, followed by an increase after transfer to darkness (Figure 6 and Figure 7A). These changes indicate that the pigment-associated photosynthetic apparatus was also affected during the light period and subsequently recovered during darkness. Strong illumination can result in excess excitation energy and formation of reactive oxygen species, which can damage chlorophyll-containing photosynthetic complexes and interfere with PSII repair, including D1 synthesis [49]. High-light acclimation of *Synechocystis* is also associated with changes in photosystem stoichiometry, chlorophyll allocation, and expression of genes involved in photosynthesis and light harvesting [50,51,52]. Consequently, the decrease in chl *a* content during prolonged illumination may be associated with photooxidative pigment damage and acclimatory remodeling of the photosynthetic apparatus. The recovery observed during the dark period may also be associated with broader temporal regulation of cellular metabolism (Figure 7A). Previous studies have shown that photosynthesis, carbon metabolism, metal transport, and energy-related pathways are extensively regulated over L/D cycles in *Synechocystis* [53]. Cyanobacterial clock-related proteins also contribute to the coordination of carbon and nitrogen metabolism during light-to-dark transitions [54]. Thus, the dark period can be considered a physiological phase associated with the repair of photosynthetic components, redistribution of cellular resources, and metabolic reorganization before the subsequent period of illumination.

The comparison between continuous illumination at 370 and 150 µmol photons m^−2^ s^−1^ further illustrates the trade-off between photon supply and photostress. Lowering the continuous irradiance to 150 µmol photons m^−2^ s^−1^ prevented the severe decline in photosynthesis observed at 370 µmol photons m^−2^ s^−1^, indicating that the extent of cumulative light stress was reduced. However, O_2_ evolution at 150 µmol photons m^−2^ s^−1^ remained considerably below that of either L/D condition because of the lower photon input. Thus, simply decreasing continuous irradiance can improve photosynthetic stability but does so at the expense of productivity.

Collectively, these results indicate that sustained photosynthetic productivity requires an appropriate balance between sufficient photon supply and opportunities for physiological recovery. Among the conditions examined, the rectangular L/D pattern provided the most favorable balance: the constant daytime irradiance avoided the high transient irradiance of the simulated solar profile, while the regular dark period allowed recovery from light-induced physiological stress. Consequently, temporal control of light delivery, rather than optimization of irradiance or total daily photon input alone, should be considered an important parameter for maintaining stable photosynthesis and growth during continuous cultivation of *Synechocystis* in photobioreactors.

## 5. Conclusions

This study demonstrates that the temporal distribution of light is an important operational parameter for maintaining photosynthetic performance in *Synechocystis* sp. PCC 6803 under turbidostatic continuous cultivation. Importantly, differences in photosynthetic stability and growth were observed even when the same daily photon input was supplied, indicating that optimization of illumination should consider not only the total amount of light delivered but also how that light is distributed over time.

Among the conditions examined, the rectangular light/dark (L/D) pattern provided the most favorable balance between photon supply and physiological recovery. In contrast, continuous illumination at 370 µmol photons m^−2^ s^−1^ resulted in a progressive decline in photosynthetic performance. The accompanying changes in D1 protein abundance and chlorophyll *a* further support the view that dark periods contribute to recovery from light-induced stress.

A major strength of this study is the direct comparison of distinct temporal illumination patterns under controlled continuous-culture conditions while maintaining comparable daily photon inputs. These results highlight temporal light management as a practical strategy for improving the stability of cyanobacterial photobioreactor operation. Rather than maximizing irradiance alone, illumination strategies that balance efficient photon delivery with periodic opportunities for physiological recovery may provide a more effective approach for sustaining biomass productivity. Further studies using additional cyanobacterial strains, cultivation scales, and dynamically optimized L/D regimes will be required to determine the broader applicability of this strategy.

## Data Availability

The raw data supporting the conclusions of this article will be made available by the authors on request.

## Supplementary Materials

The following supporting information can be downloaded at: https://www.mdpi.com/article/10.3390/microorganisms14092044/s1, Figure S1: Photograph of the flat-panel photobioreactor used for the cultivation of *Synechocystis* sp. PCC 6803 under the different illumination conditions investigated in this study.
