# Supplementary material for: Effects of the Pattern of Light and a Dark Recovery Period on Photosynthesis and Growth in Synechocystis sp. PCC 6803
Source: Microorganisms. 2026 Sep 14;14(9):2044. doi: 10.3390/microorganisms14092044 (PMC13609035; doi:10.3390/microorganisms14092044)
Supplement: Supplementary file 1 [file microorganisms-14-02044-s001.zip › microorganisms-4489107-supplementary.pdf]

Supplementary File

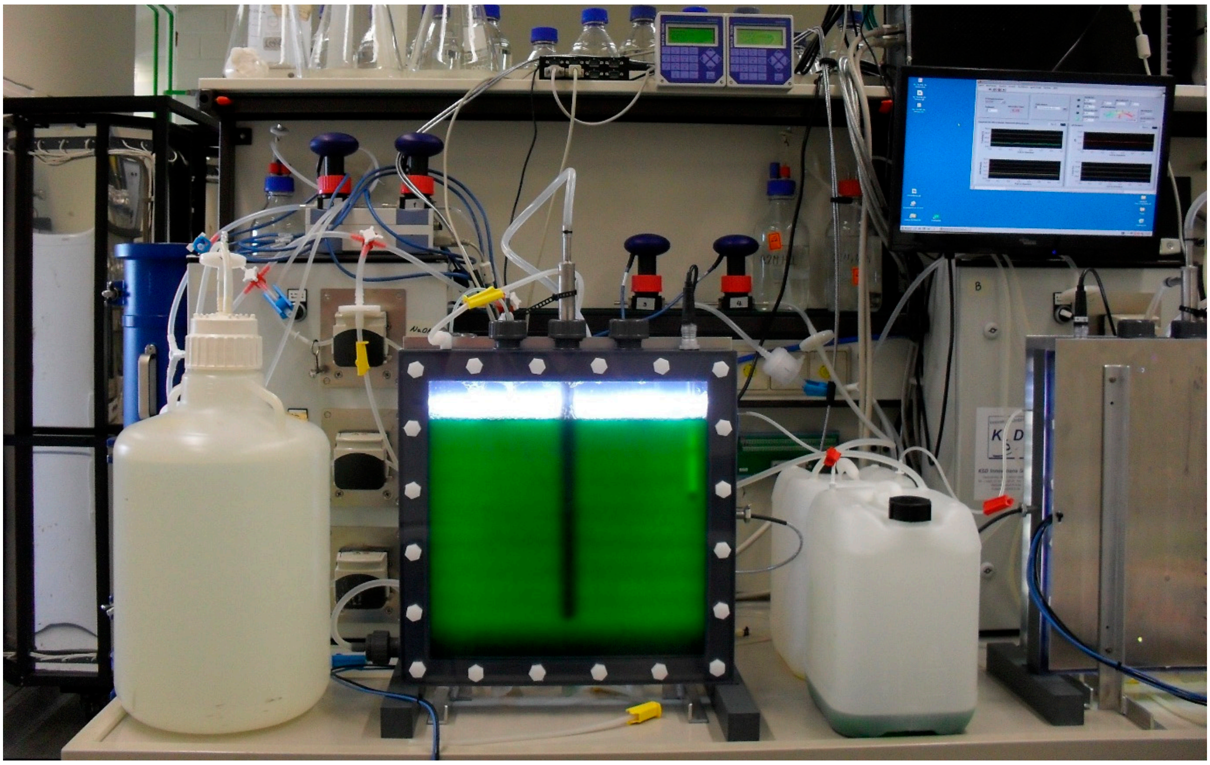

Supplementary Figure S1. Photograph of the flat-panel photobioreactor used for the cultivation of *Synechocystis* sp. PCC 6803 under the different illumination conditions investigated in this study.
